# Psychological impact of anti-VEGF treatments for wet macular degeneration—a review

**DOI:** 10.1007/s00417-016-3384-0

**Published:** 2016-06-04

**Authors:** Hugo Senra, Zaria Ali, Konstantinos Balaskas, Tariq Aslam

**Affiliations:** 1 Institute of Human Development, University of Manchester, Oxford Road, Manchester, M13 9PL UK; 2 Manchester Royal Eye Hospital, Manchester, UK; 3 Heriot Watt University, Edinburgh, UK

**Keywords:** Wet age-related macular degeneration, Anti-VEGF treatment, Psychological impact, Intra-vitreal injections

## Abstract

**Purpose:**

To review the current literature on the psychological impact of anti-VEGF treatments for wet age-related macular degeneration (wAMD), in terms of patients’ experiences of receiving these treatments, and the impact of these treatments for patients’ mental health and quality of life.

**Methods:**

We critically analyzed current literature evaluating psychological impact of anti-VEGF treatments for wAMD. Primary searches of PubMed, Science Direct, and Web of Science were conducted in July and August of 2015. We reviewed all papers on the topic published until August 5, 2015.

**Results:**

Our literature search found 14 papers addressing the psychological impact of anti-VEGF treatments for wAMD. Results highlighted potential anxieties and experiences of pain caused by receiving regular intravitreal injections. A positive visual outcome of anti-VEGF therapy is associated with positive vision-related QOL outcomes, although such association seems to be dependent on improvements on visual acuity. In the literature reviewed, patients receiving anti-VEGF treatments showed a prevalence rate of depression between 20 and 26 %.

**Conclusions:**

Although anti-VEGF treatments can cause some anxiety and being experienced as a stressful event, especially in the beginning of the treatment, preliminary findings suggest a potential benefit for long-term vision-related quality of life. Further longitudinal and qualitative research should bring more evidence on the positive and negative effects of these treatments on patients’ long-term mental health.

## Introduction

Age-related macular degeneration (AMD) is currently the leading cause of irreversible vision loss and blindness in people aged 50 and older, particularly in the developed world [1]. AMD can be a highly disabling condition, causing impairment to the activities of daily living, invoking emotional distress, anxiety, and depression [2]. Currently, wet macular degeneration (wAMD) is the only form of AMD that is treatable, usually involving the use of vascular endothelial growth factor inhibitors (anti-VEGF) such as ranibizumab, aflibercept, or bevacizumab [3]. These treatments are regarded as having great potential for halting disease progression and for reducing further risk of blindness [3].

However, these treatments are administrated by invasive intra-vitreal injections, often at the conclusion of lengthy, frequent, and oft-repeated visits after transport to an eye clinic. The specific act of an intraocular anti-VEGF injection can be experienced by patients as a stressful event, especially in the beginning of treatment [4, 5]. Furthermore, anti-VEGF treatments are frequently without a pre-established date for their completion, and this concept can entail additional anxiety to some patients.

In order to optimize patients’ quality of life, it would seem prudent to not only target the impact of treatments on vision but also the impact of the repeated injection visits themselves on patients’ well-being and mental health. It is therefore crucial to understand how patients experience these invasive treatments and how they manage any related anxieties. Additionally, it is relevant to investigate the separate issue of whether the protective effect that these treatments have on vision is also followed by any benefits on patients’ mental health and quality of life, or if the treatment benefits are exclusive to patients’ vision. Both questions are of importance for everyday clinical management of these patients, but unfortunately both are still poorly answered due to paucity of relevant research.

## Methods

With this review, we want to critically analyze the current literature on the psychological impact of anti-VEGF treatments for wAMD. We selected all studies that fulfilled the following inclusion criteria: articles published in English; peer-reviewed articles; studies of adults with wAMD; studies addressing anti-VEGF treatments for wAMD; studies focused on the psychological and psychosocial implications of receiving anti-VEGF treatments for wAMD including the experience of receiving treatment, quality of life, anxiety, stress, and depression. We only considered studies investigating psychological consequences of receiving anti-VEGF injections for wAMD. Studies addressing the psychological impact of wAMD without controlling for intravitreal injections or studies conducted with patients who did not receive these treatments were excluded. We also excluded studies exclusively focused on pain levels induced by anti-VEGF injections or on factors associated with pain caused by injections because we do not consider pain as a psychological variable and therefore it cannot be regarded as part of the psychological impact of anti-VEGF treatment. Systematic reviews were considered in this review as they summarize pre-existent literature and evidence on the topic.

Two authors (H.S. and Z.C.A.) systematically conducted a search of electronic databases (PubMed, Web of Science, and Science Direct) to retrieve all articles published up to August 5, 2015. We searched these databases using terms that are often used in literature to designate AMD, anti-VEGF treatments and its psychological impact, including “macular degeneration” and “anti-VEGF”; “macular degeneration” and “psychological”; “macular degeneration” and “depression”; “macular degeneration” and anxiety”; “anti-VEGF” and “experience”; “anti-VEGF” and “psychological”; “anti-VEGF and “depression”; “anti-VEGF” and “anxiety”; “anti-VEGF” and “quality of life”; “intravitreal” and “anxiety”; “intravitreal” and “depression”; “intravitreal” and “psychological”; “intravitreal” and “quality of life”. Additional sources were identified through cited and citing articles.

Two authors (H.S. and Z.C.A.) independently reviewed titles and abstracts and then the full-text articles to identify the eligible studies. Results of both researchers were compared, and clearly non-eligible studies were excluded. Then, duplicates were removed from the list. Next, the same researchers read the abstracts of the remaining articles to determine whether they met inclusion criteria. Abstracts providing sufficient detail for exclusion were removed, and the remaining full-text articles were retrieved. Full-text articles were read to determine inclusion, and disagreements were resolved via consensus and returning to the articles. Data were analyzed and summarized using a specific table (Table 1).

**Table 1:** Studies on psychological impact of anti-VEGF treatments for wAMD

Reference	Topic	Year of publication	Type of anti-VEGF treatment	Study design	No. of patients	Outcome measure/s	Key findings
Boyle et al. [4]	Experience of receiving anti-VEGF treatment	2014	Studies reviewed used ranibizumab and bevacizumab	Systematic review	Total number of patients from all studies reviewed - 582	Reasons for pain/discomfort Additional factors influencing level of pain Reasons for patient fear associated with undergoing anti-VEGF treatment	Anticipated discomfort is often greater than actual discomfort. Different stages of the procedure produce varying levels of patient discomfort Pain experienced varies with demographics, change in VA, and number of previous injections Common reasons include the thought of having an injection, fear of losing eyesight and fear of the unknown
McCloud et al. [5]	Experience of receiving anti-VEGF treatment	2014	Not stated	Qualitative	34	Patients experience via qualitative interviews	Four major themes arose from the narratives of the participants: cautious optimism, enduring, adaptation, and profound loss
Chua et al. [6]	Patients’ perspective of anti-VEGF treatment for wAMD	2009	Ranibizumab	Prospective clinical survey	100	A validated questionnaire covering the levels of discomfort, anxiety and fear	53 % of patients reported that anti-VEGF injections were a less fearful experience than they expected The great majority of patients were neither anxious nor fearful after the first injection
Henriksen and Adhami [7]	Experience of receiving anti-VEGF treatment	2010	Bevacizumab	Qualitative	10	Interviews relied on one open question and analyzed based on Giorgi’s phenomenological analysis	Findings highlighted four major problem areas: information; fear / discomfort; subjective expectations regarding vision; confidence in the care. These areas were associated with safety and treatment, previous experiences and fears of pain and losing eyesight
Tailor et al. [8]	Experience of receiving anti-VEGF treatment	2011	Ranibizumab	Prospective consecutive case series	42	Specific questionnaire designed to capture the detail of patients’ experiences of anti-VEGF injections	Patients reported different levels of discomfort according to the stage of the intravitreal injection procedure Needle entry, drape application, cutting of drape, insertion of speculum and waiting for injection were the stages of injection procedure that patients scored as more unpleasant and with higher discomfort
Thetford et al. [9]	Experience of receiving anti-VEGF treatment	2013	Ranibizumab	Qualitative	22	Qualitative narrative interviews using the Biographical Narrative Interpretative Method (BNIM) using a single opening question	Findings highlighted four key themes: anxiety and fear of the unknown; the injection procedure; side effects; and service delivery Needle entry, instillation of eye drops, and removal of the surgical drape were the patients’ most frequent causes of discomfort
Wang et al. [10]	Impact of anti-VEGF on quality of life	2015	Ranibizumab	Observational non-interventional study	Total number of patients initially- 80 3-month follow-up - 77	NEI-VFQ scores at baseline and at 3 months	Better visual function scores were associated with higher scores on the overall NEI VFQ-25
Finger et al. [11]	Impact of anti-VEGF on quality of life	2013	Ranibizumab	Observational non-interventional study	Total number of patients initially - 3470 4-month follow-up - 3124 12-month follow-up - 2587	Patients’ VRQoL using the NEI-VFQ	It was found that the improvements in BCVA and VRQoL could not be maintained at the 12-month follow-up
Finger et al. [12]	Impact of anti-VEGF on quality of life	2014	Ranibizumab	Observational non-interventional study	Total number of patients initially - 169 6-month follow-up - 138 12-month follow-up - 120	The VRQoL as measured by the IVI using its three subscales: Accessing Information, Mobility Emotional Well-being	Improvement in VA lead to an improvement of self-reported ability to read and access information, as well as emotional well-being A change in VA of the treated eye directly influenced the patients VRQoL irrespective of whether the better or worse eye was treated
Inoue et al. [13]	Impact of anti-VEGF on quality of life	2014	Ranibizumab	Observational non-interventional study	3-month follow-up - 54 patients 12-month follow-up −49 patients	LogMAR visual acuity and NEI VFQ-25 scores preoperatively and postoperatively	IVR treatment resulted in a higher postoperative NEI VFQ-25 score Improved visual acuity at 12 months was associated with a greater improvement in NEI VFQ-25
Casten et al. [14]	Depression after receiving anti-VEGF	2010	Not stated	Observational non-interventional study	51	NEI-VFQ subscale score for near and distance vision at baseline and 3 months later Subjective opinion of how helpful injections and obstacles to treatment	20 % of patients had clinically significant depressive symptoms Those with depression were found to have a greater decline in vision Depression was unrelated to changes in NEI-VFQ or obstacles to treatment
Lee et al. [15]	Depression after receiving anti-VEGF	2013	Ranibizumab	Cross-sectional	107	Prevalence of depression using geriatric depression scale	The prevalence of depression was 26.2 % with AMD It was suggested that age be the most important factors associated with depression in AMD. With older age, the severity of depression also increases
Sloan et al. [16]	Depression after receiving anti-VEGF	2014	Ranibizumab and bevacizumab	Longitudinal	Sample size for depression anti-VEGF therapy group (*n* = 13,258) No treatment control group (*n* = 13,258)	Number of patients newly diagnosed with depression during the follow-up period (measure or method not stated) Need for admission to a long-term care facility	A new diagnosis of depression during the follow-up period was found to be 2 %. There was no statistical difference between those who had anti-VEGF treatment, and those who did not
Segal et al. [17]	Anxiety related to receiving anti-VEGF	2015	Bevacizumab	Observational, non-interventional study	225	Pre-procedural anxiety using VASA Post procedural pain using VAS	Positive correlation between increased pre-procedural anxiety and perceived pain Correlation between surgeon and perceived pain in intravitreal injections

## Results

**Fig. 1:**
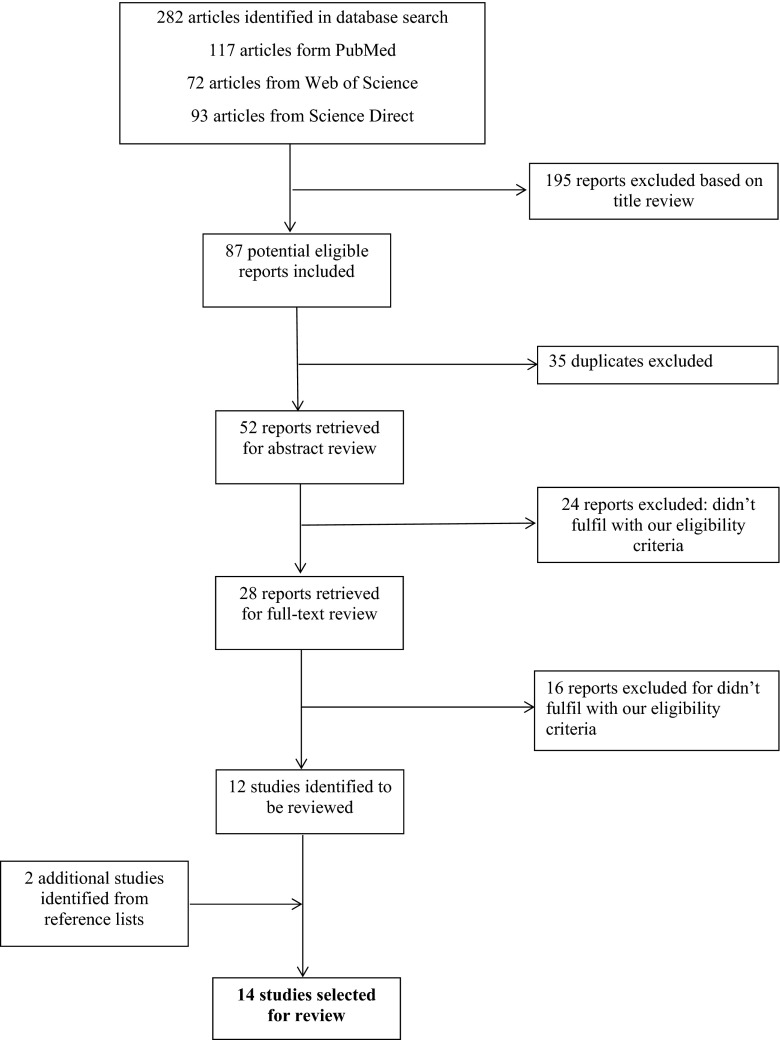
Flow diagram of articles selection for review

Figure 1 describes the process of study selection. We identified 282 articles from the databases, of which 195 were excluded on the basis of title review, leaving 87 articles. Of 87 articles, we excluded 35 for being duplicates and 40 for not meeting our eligibility criteria, with 12 articles remaining for review. Reference lists were reviewed using the same process, and two additional articles were identified for inclusion, leaving a final list of 14 articles to be reviewed [4–17]. The main reasons for not meeting the eligibility criteria of this review were samples not exclusively composed of patients with wAMD, studies about the psychological impact of anti-VEGF in other medical conditions, or studies exclusively focused on other consequences of wAMD such as pain or on the effect of anesthetics on pain levels experienced by patients.

Table 1 summarizes all articles included in the current review. The year of publication ranges from 2009 to 2015. Six articles reported observational non-interventional studies [10–14, 17] and three articles reported qualitative studies [5, 7, 9]. The remaining articles reviewed were one systematic review [4], one prospective clinical survey [6], one prospective cases series case series study [8], one cross-sectional study [15], and one longitudinal study [16]. With regards to the type of anti-VEGF treatment received by patients, ranibizumab was used in eight studies [6, 8–13, 15], while bevacizumab was only used in two studies [7, 17]. One study used ranibizumab and bevacizumab [16] and two other studies did not state which type of anti-VEGF had been administrated to patients [5, 14]. The systematic review reported studies in which ranibizumab and bevacizumab were used [4]. Studies included in this review investigated the psychological impact of anti-VEGF treatments by exploring one of the following topics: (1) Patients’ experiences or perspectives of the process of receiving anti-VEGF treatments for wAMD [4–9]; (2) Longer-term impact of anti-VEGF treatments on patients’ quality of life [10–13]; and (3) Depression/anxiety after receiving anti-VEGF treatments for wAMD [14–17].

### Patients’ experiences of the process of receiving anti-VEGF treatments for wAMD

Our review found six articles on patients’ experiences or perspectives of receiving anti-VEGF treatments for wAMD [4–9]. Findings generally highlighted frequent experiences of discomfort and fear associated with receiving anti-VEGF treatment over time [4, 5, 7–9]. The aspects of the treatment in which patients reported more discomfort were: needle entry; application of drops; insertion of speculum; waiting for injection; experiences of pain, fear of losing sight, fear for the unknown, and side effects. The most recent study we found on patient experiences of anti-VEGF highlighted the complexity and diversity of patients’ experiences of treatment. This study suggested, for the first time, patients’ “cautious optimism” as a valid response to treatment success. However, one study found that discomfort and fearful experiences were mainly circumscribed to the first injection and strongly related to the patients’ previous expectations of treatment [6]. Finally, the systematic review on the experiences of anti-VEGF treatments [4] included four studies that were also analyzed by us [6–9] and six other studies focused on pain levels caused by anti-VEGF treatment and the effect of anesthetics on pain experienced by patients [8, 18–22]. Findings highlighted the fact that patients’ expectations of pain before they started the treatment were higher than the pain experienced when receiving the treatment. Finally, the review suggested the need for more research, particularly to clarify the reasons underlying patients’ anxieties when receiving anti-VEGF treatments, and more qualitative studies addressing patients’ experiences of receiving the treatments.

### Longer-term impact of anti-VEGF treatments on patients’ quality of life

Studies reviewed found an improvement on patients’ vision-related quality of life after they have started receiving intravitreal treatment with ranibizumab for wAMD [10–13]. This relationship was found to be very dependent on improvements in patients’ visual acuity [10–13]. One study found an improvement on patients’ VRQL at month 3 of treatment [10] and another study found improvements at month 4 of treatment [11]. Two other studies found better vision-related quality of life after 12 months of treatment [11, 13]. Of four articles identified, three used the National Eye Institute Visual Function Questionnaire (NEI VFQ-25) [23] to assess patients’ vision-related quality of life. This instrument comprises the assessment of general health, quality of vision, and vision-related quality of life that includes dependency, role limitations, mental health, social functioning, ocular pain, and driving. In general these studies found an improvement on patients’ vision-related quality of life after they have started receiving intravitreal treatment with ranibizumab for wAMD. An improvement on patients’ mental health was also associated with receiving these treatments [10–13].

### Depression and anxiety after anti-VEGF treatments for wAMD

A preliminary study has explored depression in wAMD patients receiving anti-VEGF treatments [14]. In this study, depression was assessed using the NEI VFQ [23]. Results suggested a slightly lower prevalence rate of depression among patients receiving anti-VEGF treatments in comparison with previous studies in which depression rates were not adjusted for anti-VEGF treatments. Additionally, patients with depression were found to have a greater decline in vision. Finally, depression was unrelated to changes in NEI-VFQ or obstacles to treatment.

With different results, a study conducted with 107 Korean patients with wAMD receiving intravitreal ranibizumab treatment found a prevalence of depression of 26.2 %, which is consistent with the literature pre-anti-VEGF treatments [15]. In this study, depression was assessed using the Geriatric Depression Scale [24].

A retrospective study analyzed the incidence of a primary diagnosis of depression among Medicare beneficiaries in the US diagnosed with wAMD during a 2-year follow-up period [16]. In this study, the diagnosis of depression was identified from the enrolment information and Medicare claims filed on behalf of beneficiaries. This study reported no significant differences in the incidence of depression between patients receiving anti-VEGF treatments and those who did not. Furthermore, a first diagnosis of depression during the follow-up period only occurred in 2.0 % of the whole sample.

Finally, a recent prospective observational study conducted in Israel found a significant correlation between patients’ anxiety levels experienced before the injection and pain experienced when receiving the injection [17]. In this study, 25 % of the participants reported high levels of anxiety measured by a visual analogue scale (score ≥ 6 on a scale from 0 to 10).

## Discussion

Our review shows that the psychological impact of anti-VEGF treatments for wAMD remains a relatively new topic with limited evidence and therefore requires more research. To date, the psychological impact of receiving anti-VEGF treatments for wAMD has been addressed in studies focused on the patient experience of receiving these treatments, the impact of anti-VEGF treatments for patients’ vision-related quality of life, and mental health.

The patient experience of receiving anti-VEGF has been the most addressed topic in previous studies about the psychological impact of these treatments for wAMD. In this topic, we found five articles [5–9] and one systematic review in which ten articles were reviewed [4]. Findings suggested that, in general, this treatment is well tolerated by patients, but a great portion of them were still anxious about the treatment. According to these studies, patients feel anxious especially because of previous expectations of receiving a needle in the eye, fear of losing their sight, fear of any side effects, and prior experiences of pain when receiving intravitreal injections. Furthermore, as a recent study highlighted [5], anti-VEGF treatments can have significantly changed the way patients experience this disease and cope with the fear for blindness, because now patients come across with a treatment with great potential for halting disease progression. Studies conducted prior to availability of anti-VEGF treatments considered a patients’ optimism about the medical treatments and disease progression as false hope and a non-adapted behavior [25], but patients’ cautious optimism about the disease progression can now be acceptable in the light of positive outcomes offered by anti-VEGF treatments and the way the wAMD prognosis is now communicated to patients [5]. However, there are some aspects of the experience of receiving anti-VEGF treatments needing more attention. In this review, we only found three studies exploring in-depth patients’ experiences of receiving anti-VEGF treatments using a qualitative design [5, 7, 9]. The remaining studies that have addressed the experience of receiving anti-VEGF treatment were mainly focused on pain caused by the treatment. It is of paramount importance to generate more evidence on how patients experience these treatments, especially through qualitative research, which could yield high-quality information and is a field as yet largely unexplored [4]. Such evidence is crucial to understand patients’ main sources of anxiety and what strategies patients activate to effectively cope with these treatments.

The literature we reviewed suggested that anti-VEGF treatments can have a positive impact on patients’ vision-related quality of life, albeit such a relationship is dependent on improvements in VA [10–13]. In these studies, the concept of vision-related quality of life included not only a patient’s aspects related to the use of vision in activities of everyday life but also mental health and social functioning. These findings therefore suggested a strong link between VA and patients’ perceptions of quality of life, functioning, and mental health. However, further research should clarify this link as, according to a recent systematic review, not all previous studies found a strong relationship between VA and adjustment to vision loss or depression after vision loss [26].

There is strong evidence that AMD patients are likely to be depressed and anxious as a consequence of limitations imposed by vision loss [1, 2, 27, 28]. Both conditions are regarded as the most frequent mental health problems among people with AMD with rates ranging from 15.7 to 44 % for depressive symptoms and 9.6 to 30.1 % for anxiety symptoms [2]. Depression would be an additional source of disability for these patients and can compromise the response to the treatment and medical outcomes [1, 2, 29]. Previous studies found high comorbidity of depression and AMD but most of them did not control for patients who were receiving anti-VEGF treatments [20, 29]. It is important to consider the potential influence of anti-VEGF treatments on prevalence of depression because some studies have suggested a positive correlation between visual acuity and adjustment to vision loss [26–28], mental health [10–13], and quality of life [10–13]. In our review, we found three studies addressing depression after receiving anti-VEGF treatments for wAMD [14–17]. Two of them suggest no influence of anti-VEGF treatments on prevalence rates of depression. However, in one of these studies [15], findings should be carefully analyzed because: (a) the authors did not provide any details on how depression was measured and diagnosed during the follow-up period; and (b) the incidence rates of depression found in this study are oddly lower than those found in previous studies in which standardized and valid instruments were used to measured depression [2]. Finally, the only study that assessed the levels of anxiety associated with receiving anti-VEGF injections suggested that anti-VEGF treatment can induce clinical anxiety, and that the levels of anxiety found are within the range of prevalence of anxiety described in previous studies evaluating psychological impact of AMD [2].

Some limitations should be acknowledged in this review. First, only articles in English were included in this review, which can be considered a limitation because there are studies on the topic published in other languages. Second, the terminology used to perform the search for articles reflects not only the state of the art but also our previous experience and perhaps bias in the field. Finally, this review did not include grey literature such as academic dissertations or conference abstracts, and therefore there could be other studies addressing this topic that were not reviewed.

In conclusion, there is still very little knowledge on the psychological impact of anti-VEGF treatments for wAMD. Future studies, especially longitudinal research, should bring more evidence on this topic and clarify the positive and /or negative impact that this treatment can have for patients’ mental health and quality of life. An awareness of the likely psychological impact on individual patients should allow physicians to decide holistically, which are the most appropriate treatment strategies to adopt. Such knowledge will be key to developing evidence-based clinical strategies to help patients to manage the treatment and reduce anxiety and pain levels along the treatment. An exploration of depression in patients receiving anti-VEGF treatments would also be valuable for developing new intervention strategies to prevent long-term mental health problems among these patients. Additionally, it will be helpful to prevent any patient drop-outs, which can compromise any positive outcomes brought by anti-VEGF treatments. Because ant-VEGF treatments are being used with other ophthalmologic conditions rather than wAMD, it would be relevant to study experiences of treatment, adherence to treatment, mental health, and quality of life across other conditions such as diabetic macular edema or choroidal neovascularization secondary to pathologic myopia. The specific case of macular edema deserves special attention because there is evidence of a strong link between diabetes and depression, regardless the co-existence of vision loss [30–32].

Great advances have been made over the last decade in treating a generation of patients for whom there had previously been no hope for visual gain and further research on psychological impact of anti-VEGF treatments should expand on such advances for the overall welfare and benefit of patients with retinal disease.
